# Distribution of Gold Nanoparticles in the Anterior Chamber of the Eye after Intracameral Injection for Glaucoma Therapy

**DOI:** 10.3390/pharmaceutics13060901

**Published:** 2021-06-17

**Authors:** Tobias Sonntag, Franziska Froemel, W. Daniel Stamer, Andreas Ohlmann, Rudolf Fuchshofer, Miriam Breunig

**Affiliations:** 1 Department of Pharmaceutical Technology, University of Regensburg, Universitaetsstrasse 31, 93040 Regensburg, Germany; tobias.sonntag@chemie.uni-regensburg.de; 2 Department of Human Anatomy and Embryology, University of Regensburg, Universitaetsstrasse 31, 93040 Regensburg, Germany; franziska.froemel@vkl.uni-regensburg.de (F.F.); rudolf.fuchshofer@vkl.uni-regensburg.de (R.F.); 3 Department of Ophthalmology, Duke University, Durham, NC 27710, USA; william.stamer@dm.duke.edu; 4 Department of Ophthalmology, Ludwig-Maximilians-University Munich, Mathildenstrasse 8, 80336 Muenchen, Germany; andreas.ohlmann@med.uni-muenchen.de

**Keywords:** glaucoma, gold nanoparticles, anterior chamber, distribution, stability, intracameral injection, trabecular meshwork, hyaluronic acid

## Abstract

In glaucoma therapy, nanoparticles (NPs) are a favorable tool for delivering drugs to the outflow tissues of the anterior chamber of the eye where disease development and progression take place. In this context, a prerequisite is an efficient enrichment of NPs in the trabecular meshwork with minimal accumulation in off-target tissues such as the cornea, lens, iris and ciliary body. We evaluated the optimal size for targeting the trabecular meshwork by using gold NPs of 5, 60, 80 and 120 nm with a bare surface (AuNPs) or coated with hyaluronic acid (HA-AuNPs). NPs were compared regarding their colloidal stability, distribution in the anterior chamber of the eye ex vivo and cellular uptake in vitro. HA-AuNPs demonstrated an exceptional colloidal stability. Even after application into porcine eyes ex vivo, the HA coating prevented an aggregation of NPs inside the trabecular meshwork. NPs with a diameter of 120 nm exhibited the highest volume-based accumulation in the trabecular meshwork. Off-target tissues in the anterior chamber demonstrated an exceptionally low gold content. Our findings are particularly important for NPs with encapsulated anti-glaucoma drugs because a higher particle volume would be accompanied by a higher drug payload.

## 1. Introduction

With an estimated 60.5 million cases, glaucoma is one of the leading causes of irreversible blindness worldwide [1]. Primary open-angle glaucoma (POAG) is the most prevalent form of glaucomatous diseases [2]. Eye drops are applied as first-line treatment to reduce the intraocular pressure (IOP), which is the major risk factor of the disease. However, bioavailability of topically applied drugs in the anterior chamber is only about 1 to 5% [3]. Another fundamental problem is that most conventional drugs do not target the causal pathological changes occurring in the outflow tissues [4]. Here, the trabecular meshwork and Schlemm’s canal are affected by an increased production of extracellular matrix and a significant stiffening of cells [4]. Therefore, it is crucial to develop new causative options to drastically improve the therapeutic outcome. Recently, new drugs have been approved that interfere with the fundamental pathological processes of POAG development, such as Rho-associated protein kinase inhibitors (e.g., netarsudil and ripasudil) [5] or nitric oxide donors (e.g., latanoprostene bunod) [6]. In addition to these small-molecule drugs, macromolecules, such as small interfering RNA (siRNA), are discussed as an innovative alternative to intervene disease progression [7,8]. However, their high molecular weight, inherent instability and strong negative charge currently prevent nucleic acids to reach a sufficiently high bioavailability in the anterior chamber of the eye [9]. 

To significantly boost the availability of drugs in the outflow tissues, we and others recently proposed nanoparticles (NPs) as a delivery system to the anterior eye [10,11,12]. NPs are expected to follow the physiological outflow pathway of the aqueous humor, thereby reaching the trabecular meshwork and Schlemm’s canal [13]. Aqueous humor is secreted by the ciliary epithelial cells and drains from the posterior to the anterior chamber. A minor part exits the eye through the uveoscleral pathway, while the major part of the aqueous humor leaves the eye through the trabecular meshwork into Schlemm’s canal [14]. The outflow resistance, responsible for the IOP, is located within the juxtacanalicular tissue and the endothelium of Schlemm’s canal [14]. The cells of this tissues represent the target cells of the NPs. Once arrived at the target cells, NPs have the great advantage that they allow for an efficient cellular uptake of their therapeutic freight [15]. Macromolecular drugs in particular, such as siRNA, would otherwise be highly unstable and not be able to cross cellular membranes [16]. Different types of NPs for intracameral injection have been evaluated, such as inorganic silica NPs [17] or polymer NPs like poly(lactic-co-glycolic acid) (PLGA) [10]. A critical parameter is the size of the particles as it influences their transport through the tissue and uptake into target cells [18]. However, in contrast to other tissues [19], there has been no systematic approach to determine which NP size may be optimal to maximize the amount of drug that arrives at its final destination within the anterior chamber of the eye. A detailed characterization of the specific NP features would be of utmost importance to create a specific and effective drug delivery system that targets the outflow tissues. Equally important is if the NPs are eventually distributed to off-target tissues in the anterior eye, such as the cornea, lens, iris or ciliary body.

Therefore, in this study, we systematically examined the size-dependent distribution of NPs in the anterior eye, with a particular focus on the outflow tissues after intracameral injection. Model gold NPs of different sizes of 5, 60, 80 and 120 nm were evaluated. Particles with a bare surface (AuNPs) were compared to gold NPs that were modified with hyaluronic acid (HA-AuNPs). Hyaluronic acid (HA) was chosen because it may improve the colloidal stability of the NPs and because it is a widely used excipient in ocular dug delivery [20,21]. The surface- and size-dependent distribution of the NPs after intracameral injection was examined after ex vivo perfusion of porcine eyes. Additionally, to evaluate the successful cellular uptake of the NPs, in vitro cell culture experiments were performed.

## 2. Materials and Methods

### 2.1. Materials

Spherical AuNPs with a citric acid coated surface and a size of 5, 60, 80 and 120 nm were purchased from Nanopartz (Loveland, CO, USA). Dulbecco’s modified Eagle’s medium (DMEM) containing 4.5 g/L glucose and 1 g/L glucose for cell culture was obtained from Merck (Darmstadt, Germany) and fetal bovine serum (FBS) was bought from Biowest (Nuaillé, France). Dialysis membranes (molecular weight cut-off = 25,000 and 3500 Da) were purchased from Carl Roth (Karlsruhe, Germany) and sodium hyaluronate (HA; 13 kDa) from Lifecore Biomedicals (Chaska, Minnesota, USA). All other chemicals were purchased from Merck (Darmstadt, Ger-many). 3-(4,5-Dimethylthiazol-2-yl)-2,5-diphenyltetrazolium bromide (MTT) was obtained from PanReac AppliChem (Darmstadt, Germany).

### 2.2. Cell Culture

Primary human trabecular meshwork (hTM) cells and Schlemm’s canal (SC) cells were fully characterized according to standard methods (PMIDs: 9727403, 29526795) and used until passage number 8. Primary fibroblasts were used until passage 13. All procedures for collecting human tissue were according to the Declaration of Helsinki. hTM cells, SC cells and fibroblasts were cultivated in DMEM (supplemented with 4.5 g/L glucose for fibroblasts and 1 g/L glucose for hTM and SC cells) containing 10% (*v*/*v*) FBS, 100 units/mL penicillin and 100 µg/mL streptomycin. Immortalized human TM cells (HTM-N) were obtained from Iok-Hou Pang and Louis DeSantis (Alcon Research Laboratories, Fort Worth, TX, USA). They were cultured in DMEM high glucose (4.5 g/L) with L-glutamine (0.584 g/L) and 1 mM sodium pyruvate. Human umbilical vein endothelial cells (HUVECs) were grown in Endothelial Cell Growth Medium MV + supplements (PromoCell, Heidelberg, Germany) with 10% FBS, 100 units/mL penicillin and 100 µg/mL streptomycin.

### 2.3. Preparation of HA-Modified AuNPs

Preparation of thiolated HA: Thiol end-modified HA (HA-SH) was prepared by reductive amination as previously described [22]. In brief, 100 mg HA (13 kDa) and 60 mg cystamine dihydrochloride were dissolved in 10 mL 0.1 M borate buffer (pH = 8.5) containing 0.4 M NaCl and stirred for 2 h. NaBH_3_CN was added to the solution for a final concentration of 200 mM and stirred at 40 °C for 5 days. Afterwards, 100 mM dithiothreitol was added and stirred again for 12 h. The resulting HA-SH was dialyzed using a dialysis membrane (MWCO = 3500 Da) against 5 L of 100 mM NaCl solution for 2 days followed by 25% ethanol for 1 day and pure water for 1 day to remove the unreacted chemicals. Finally, the product was freeze-dried for 5 days. HA-SH was characterized using an Avance 300 NMR-spectrometer (Bruker Bio Spin, Ettlingen, Germany).

Preparation of AuNPs with a hyaluronic acid surface (HA-AuNPs): Thiol-gold chemistry was used for the modification of AuNPs with HA: 248 µL HA-SH solution (1 mg/mL) was added to 2 mL AuNP dispersion and stirred for 48 h. HA-AuNPs were purified using a dialysis membrane (MWCO = 25,000) against purified water for 72 h.

### 2.4. Characterization of Gold NPs

Transmission electron microscopy (TEM): To evaluate the size and the presence of aggregates, all particle species were imaged using a 100 kV Zeiss Libra 120 electron microscope (Carl Zeiss AG, Oberkochen, Germany) at a magnification of 80,000×. For analysis, samples were pipetted onto carbon-coated copper grids (300 mesh; Plano, Wetzlar, Germany) and incubated for 3 min. Excess NP dispersion was removed with a filter paper. All grids were air-dried and stored in a desiccator until TEM analysis.

Hydrodynamic diameter and zeta potential: The size and zeta potential of all NP species were measured with a Malvern Zetasizer Nano ZS (Malvern, Herrenberg, Germany). All samples were measured with a 633 nm He-Ne laser at an angle of 173° backward scatter (25 °C) in 10% Dulbecco’s Phosphate-Buffered Saline liquid (DPBS) using either polystyrene semi-microcuvettes (dynamic light scattering; Sarstedt, Nuembrecht, Germany) or folded capillary cells (zeta potential; Malvern, Herrenberg, Germany), respectively. 

UV-Vis spectroscopy: UV-Vis spectroscopic measurements were performed with a FluoStar Omega (BMG Labtech, Ortenberg, Germany). UV-Vis spectra of all particle types were taken in DPBS (λ = 400 to 800 nm).

### 2.5. Colloidal Stability of Gold NPs

For stability analysis, gold NPs were prepared as used for the in vitro and ex vivo experiments. For the in vitro stability test of gold NPs, particles were dispersed in DMEM with pyruvate containing 0.35% FBS for a final gold concentration of 2750 ppb Au. For the ex vivo stability test, gold NPs were diluted with DPBS containing 5 mM glucose for a final gold concentration of 9390 ppb Au. The hydrodynamic diameter (z-average size) was measured for every particle type after 0, 0.5, 4, 6 and 20 h.

### 2.6. Uptake of Gold NPs in Different Cell Types In Vitro

Uptake experiments were performed using all cell types and particle species. Six-well plates were used to perform the uptake experiments. All experiments were performed at pH 7.4. First, NP dispersions in DMEM containing 0.35% fetal bovine serum were prepared at a final gold concentration of 0, 550, 2750 and 5500 ppb. All samples were pre-incubated for 1 h to induce the formation of the protein corona. Afterwards, the NP dispersions were added to the cells. For the SC and hTM cells, the number of different concentrations was reduced due to the rarity of these cells. Sizes of 80 nm and 120 nm gold NPs with both surfaces were used at a concentration of c(Au) = 5500 ppb. After 6 h of incubation (37 °C, 5% CO_2_), the NP dispersion was removed, and the cells were washed twice with DPBS. Afterwards, 3 mL of aqua regia was added to each well and incubated at 60 °C for 6 h. Each sample was diluted to a final volume of 10 mL with 5% nitric acid (*v*/*v*) and Scandium as an internal standard was added. The final concentration of the internal standard was 1 µg/mL. Inductively coupled plasma–mass spectrometry (ICP-MS) measurements were performed with an ELAN 6000 (Perkin Elmer, Waltham, MA, United States). Experiments were performed in independent triplicates.

### 2.7. Perfusion Experiments of Porcine Eyes Ex Vivo

Fresh enucleated porcine eyes were obtained from a local butcher. Eyes with visible damage to the cornea were excluded. After removal of extraocular tissue, the porcine eyes were submerged into the limbus in 0.89% NaCl at 35 °C. The perfusion procedure was performed as previously described [10]. Particles were diluted in DPBS containing 5 mM glucose to a final gold concentration of 9390 ppb Au. The perfusion system consisted of a perfusion chamber and a collection chamber. The infusion needle was inserted into the anterior chamber via the cornea and connected to the perfusion chamber. The needle was moved through the pupil and the tip of the needle was placed in the posterior chamber. A second needle was positioned into the anterior chamber and connected to the collection reservoir. This drain was closed during the perfusion except during the exchange periods. First, a volume of 4 mL gold NPs (c(Au) = 9390 ppb) was exchanged over a time period of about 10–15 min. The NP concentration for ex vivo testing was based on analytical considerations and was determined using spiked tissue samples. Higher gold concentrations resulted in a non-linear increase of the signal, while lower gold concentrations revealed results below the detection limit of the ICP-MS method. Afterwards, the collection drain was closed, and the eyes were perfused with NPs at a constant pressure of 10 mmHg for 3 h. Then, a second exchange of perfusion solution with 4 mL of a 5 mM glucose solution was performed to remove the remaining NPs. Afterwards, unbound NPs were washed away by perfusion with 5 mM glucose solution for 2 h. The anterior chamber of the eye was dissected into ciliary body, cornea, iris, lens and trabecular meshwork. All tissue samples were freeze-dried for 3 days to obtain the dry weight of each tissue sample. Experiments were performed at least in independent triplicates. For the evaluation of NP distribution by electron microscopy, small samples of cornea-scleral slices, containing the trabecular meshwork, were withheld.

### 2.8. Electron Microscopy Examination of the Trabecular Meshwork

Porcine eyes were obtained from the ex vivo perfusion experiments. For TEM analysis, cornea-scleral slices from one quadrant per eye were fixed in Karnovsky’s solution (2.5% glutaraldehyde and 2.5% paraformaldehyde in a 0.1 M cacodylate buffer) for 24 h [23]. After rinsing in the 0.1 M cacodylate buffer, postfixation was accomplished in a mixture of 1% OsO_4_ and 0.8% potassium ferrocyanide in a 0.1 M cacodylate buffer for 3.5 h at 48 °C. The eyes were then dehydrated in a graded series of ethanol and embedded in Epon (Serva, Heidelberg, Germany). Semithin sections (1 µm) were collected on uncoated glass slides and stained with methylene blue/azure II (LIT). Ultrathin sections were mounted on uncoated copper grids, stained with uranyl acetate and lead citrate, and examined on a Zeiss Libra transmission electron microscope (Carl Zeiss AG). The number of NPs in the trabecular meshwork was quantified and related to an area of 1000 μm². Particles were counted in the entire trabecular meshwork and divided into the outer and inner part of the trabecular meshwork. Particles that did not penetrate deeply into the trabecular meshwork were attributed to the outer trabecular meshwork, while particles that were found deeper in the trabecular meshwork were assigned to the inner trabecular meshwork. Images were analyzed with ImageSP (TRS, Moorenweis, Germany) and QuPath (version 0.2.3, University of Edinburgh, Edinburgh, UK).

### 2.9. Gold Content of the Cells and Tissue Samples 

For ICP-MS measurements, each tissue sample was subjected to a microwave digestion for 1 h at 140 to 190 °C in aqua regia. Subsequently, each sample was diluted to 10 mL with 5% (*v*/*v*) nitric acid and Scandium as an internal standard at a final concentration of 1 µg/mL was added. ICP-MS-measurements were performed with an ELAN 6000 (Perkin Elmer, Waltham, MA, USA).

### 2.10. Statistical Analysis

All reported data were calculated as the mean ± standard deviation of at least three independent samples. When necessary, standard deviations were calculated according to the rules of propagation of errors. Unpaired, two-tailed Welch’s t-tests for statistical analyses were performed with GraphPad PRISM 6.01 software (San Diego, CA, USA) to assess statistical significance (*p* < 0.05).

## 3. Results

### 3.1. Modification and Physicochemical Characterization of Gold NPs

**Scheme 1:**
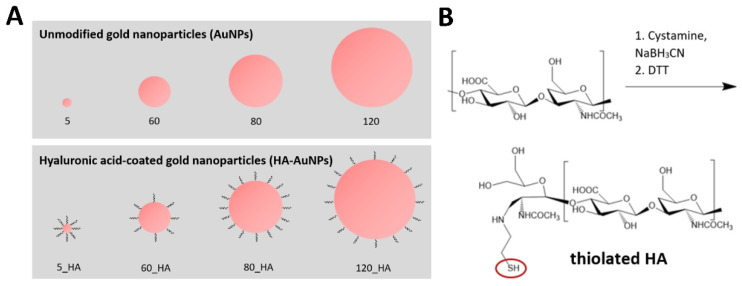
Schematic drawing of gold NPs and their modification. (**A**) For in vitro and ex vivo experiments, gold NPs with a nominal size of 5, 60, 80 and 120 nm were applied. Particles were decorated with hyaluronic acid (HA-AuNPs) to determine their physicochemical properties, cellular uptake and distribution in the anterior chamber of the eye in comparison to AuNPs with an unmodified surface. (**B**) For modification, end-thiol-modified HA was synthesized and coupled to the particles using thiol-gold chemistry.

In this study, commercially available gold NPs of different sizes were applied for cellular uptake studies in vitro and perfusion of porcine eyes ex vivo (Scheme 1A). In all experiments, unmodified AuNPs were compared to gold NPs carrying HA on their surface (HA-AuNPs).

In a first step, HA was immobilized on the surface of unmodified AuNPs. To this end, a terminal thiol group was introduced into HA, resulting in thiolated HA. The reaction was performed using reductive amination (Scheme 1B) [22]. ^1^H-NMR-spectroscopy validated the successful conversion to thiolated HA (see Supplementary Material Figure S1). Thiolated HA was then coupled to the AuNPs via thiol-gold chemistry [24], resulting in a partially covalent chemical bond between the thiol of HA-SH and the AuNP surface.

**Figure 1:**
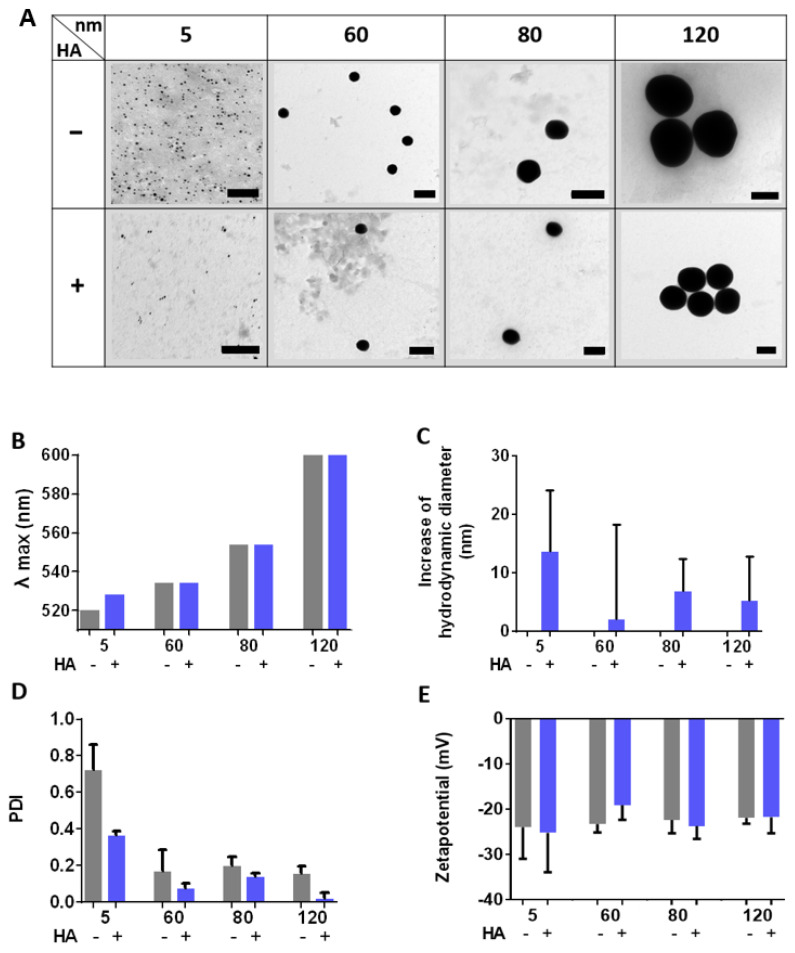
Physicochemical characterization of the AuNPs and HA-AuNPs. (**A**) TEM images demonstrated a spherical shape of all particles. The sizes of the AuNPs were 5.7 ± 3.7 nm, 60.8 ± 3.4 nm, 78.2 ± 4.6 nm and 119.0 ± 7.1 nm, respectively. The sizes of the HA-AuNPs were 5.1 ± 2.4 nm, 64.7 ± 2.5 nm, 88.6 ± 10.2 nm and 125.1 ± 9.3 nm, respectively. There was no considerable difference between the AuNPs and HA-AuNPs. The scale bar indicates 100 nm. (**B**) The SPR absorbance maximum of the particles showed a right shift with increasing particle size. For the AuNPs, the UV-maxima were 520 nm, 534 nm, 554 nm and 604 nm, respectively. For the HA-AuNPs, the UV-maxima were 528 nm, 534 nm, 554 nm and 606 nm. HA surface modification caused a right-shift of the UV-maximum only in case of the 5 nm AuNPs. (**C**) To illustrate the size increase after coating with HA, the difference in the hydrodynamic diameter between the HA-AuNPs and AuNPs was calculated. The hydrodynamic diameter differed by about 2 to 14 nm and was not statistically significantly different. (**D**) The polydispersity index (PDI) decreased with increasing size and after HA coating. (**E**) The zeta potential of all particle types was negative (−20 to −25 mV).

TEM visualized the spherical shape of all particle species. The diameters of the AuNPs were determined to be 5.7 ± 3.7 nm, 60.8 ± 3.4 nm, 78.2 ± 4.6 nm and 119.0 ± 7.1 nm, respectively (Figure 1A). The diameter of the modified HA-AuNPs was similar. The HA coating did not cause any visible changes of HA-AuNPs like aggregation. As expected, TEM did not visualize HA on the gold surface. For simplicity reasons, in the following, the AuNPs and HA-AuNPs are denoted with a size of 5, 60, 80 and 120 nm, respectively.

Gold NPs have a characteristic surface plasmon resonance (SPR) absorbance in the visible spectrum depending on their size and dielectric constant of the surrounding medium [25]. As expected, the SPR peak shifted towards larger wavelengths with increasing particle size for both AuNPs and HA-AuNPs (Figure 1B). For 5 nm AuNPs, also a shift in the SPR peak from 520 to 528 nm was determined after coating, indicating a successful deposition of HA. For other particle sizes, a shift after coating with HA was not detectable.

To verify the HA coating, the hydrodynamic diameter of the particles was determined. Values after and before coating were subtracted and expressed as size increase. As expected, the HA coating led to a slight size increase between 2 and 14 nm (Figure 1C). It seemed that particularly the 5 nm particles showed a larger size increase after coating. The polydispersity index (PDI) of the NPs decreased with increasing size (Figure 1D), which means that the size distribution of the particles became narrower. In addition, HA-AuNPs had a lower PDI compared to AuNPs. The surface zeta potential of all particle species was around −20 to −25 mV (Figure 1E).

### 3.2. Colloidal Stability of Gold NPs

**Figure 2:**
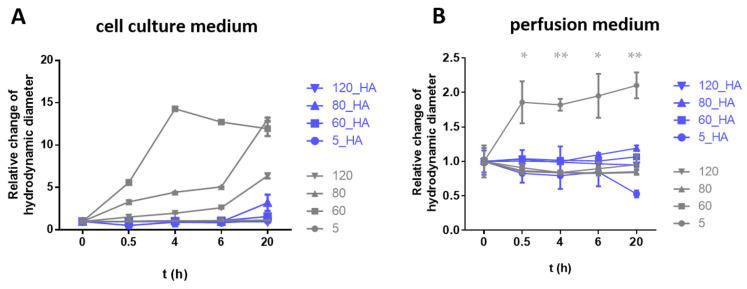
Colloidal stability of NPs in cell culture and perfusion media. The stability of AuNPs (grey lines) and HA-AuNPs (blue lines) was tested in cell culture medium (**A**) and in perfusion medium (**B**) over a time period of 20 h. The hydrodynamic diameter after certain time points was related to the initial hydrodynamic diameter (t = 0 h) and reported as the relative hydrodynamic diameter. Measurements were taken after 0, 0.5, 4, 6 and 20 h. AuNPs showed a significant increase in size after incubation in the cell culture medium. HA-AuNPs did not show an increase in size, indicating a higher stability in the cell culture medium. In perfusion medium, none of the particles showed a significant increase in size, indicating a sufficient stability. An exception was the 5 nm AuNPs. Statistically significant differences between 5 nm AuNPs and 5 nm HA-AuNPs are denoted by * or ** (*p* < 0.05 or *p* < 0.01, respectively).

Another important factor for further application is the stability of the NPs in physiological media because aggregation can significantly alter the physicochemical properties. To assess the colloidal stability, AuNPs and HA-AuNPs were incubated in cell culture or perfusion medium as it will be used subsequently either for in vitro or ex vivo studies, respectively. Then, the hydrodynamic diameter of the particles was monitored over time (Figure 2). In cell culture medium, HA-AuNPs did not show any significant change in the hydrodynamic diameter, even after 20 h of incubation, while the AuNPs showed a tremendous increase in size, indicating the presence of large aggregates (Figure 2A). In contrast, in the perfusion medium, there was no discernable difference between the AuNPs and HA-AuNPs (Figure 2B). Instead, both particle species demonstrated excellent colloidal stability in the perfusion medium with the exception of 5 nm AuNPs. The enormous size increase of 5 nm AuNPs indicated the presence of larger aggregates. To corroborate the higher colloidal stability of the HA-AuNPs, the protein adsorption on the NP surface in serum containing medium was investigated by using SDS-PAGE. HA-AuNPs revealed a significantly lower protein adsorption compared to AuNPs (see Supplementary Material Figure S2). 

### 3.3. Perfusion of Porcine Eyes with Gold NPs Ex Vivo

**Figure 3:**
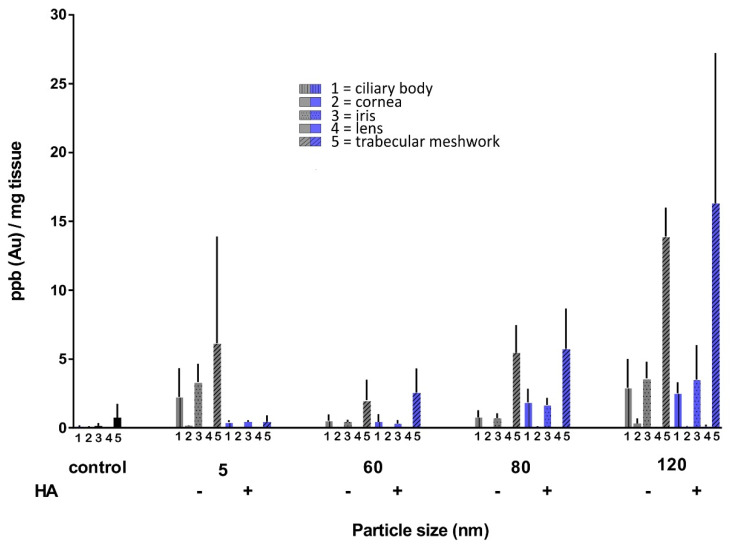
Perfusion of porcine eyes with gold NPs ex vivo. Freshly enucleated porcine eyes were used for ex vivo perfusion. Perfusion was performed with 5, 60, 80 and 120 nm AuNPs (grey bars) and HA-AuNPs (blue bars), respectively. The applied particle concentrations were normalized to a total gold concentration of 9390 ppb. Afterwards, the eyes were dissected into the ciliary body (1), cornea (2), iris (3), lens (4) and trabecular meshwork (5). Each tissue sample was dried, weighed, dissolved in aqua regia and analyzed separately. The amount of gold in parts per billion per mg tissue was analyzed by using ICP-MS. Control experiments (black bars) were conducted with perfusion medium without any gold NPs.

Ex vivo perfusion of porcine eyes was performed to determine the distribution of gold NPs in their anterior chambers. A total of 6 h after injection of the gold NPs, the anterior chamber was dissected and the ciliary body, cornea, iris, lens and trabecular meshwork were prepared. The gold content of each tissue sample was determined by ICP-MS and related to the dry weight of the tissue (Figure 3). A general trend was that with increasing particle size a higher gold content was measured. The gold content directly correlates to the NPs´ volume. This means that with an increasing size of the applied gold NPs, the overall particle volume in the tissue increased. An exception was the 5 nm AuNPs. Within one particle size and surface modification, the gold content was particularly pronounced in the trabecular meshwork and higher than in other tissues such as the cornea, iris, lens and ciliary body. In the cornea and lens, almost no gold was detected. These observations were true for all particle sizes of AuNPs and HA-AuNPs. 

**Figure 4:**
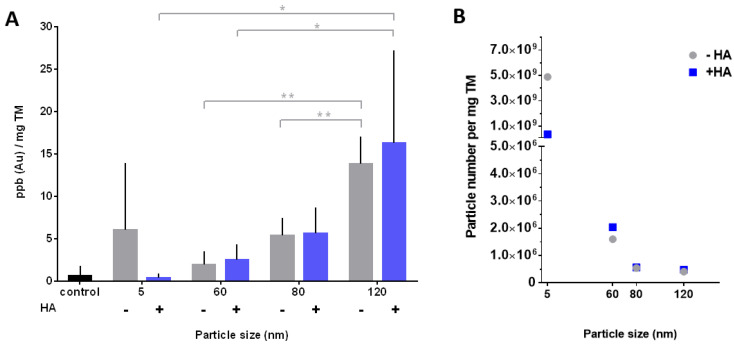
Gold content and the particle number in the trabecular meshwork (TM) after ex vivo perfusion with gold NPs. (**A**) The amount of gold per mg tissue in the trabecular meshwork was analyzed using the data from Figure 3. Grey bars represent the trabecular meshwork samples perfused with AuNPs, and blue bars represent the samples perfused with HA-AuNPs. The control is shown in the black bar. Larger particles led to a significantly higher gold content in the trabecular meshwork than smaller particles, except for the 5 nm AuNPs. Statistically significant differences are denoted by * or ** (*p* < 0.05 or *p* < 0.01, respectively). (**B**) The gold content per mg trabecular meshwork was calculated to obtain the particle number per mg trabecular meshwork. The smallest gold NPs showed the highest number of incorporated particles, but larger particles reflect the higher overall particle volume.

Because the trabecular meshwork is of particular interest, we had a closer look and directly compared the gold amount after application of the different particle types (Figure 4A). It was even more obvious that an increasing size led to a significantly higher gold content. Again, the 5 nm AuNPs did not follow this pattern. There was no statistically significant difference between the AuNPs and HA-AuNPs. Having a look at the number instead of the volume of gold NPs in the trabecular meshwork, the trend was vice versa. Then, the number of particles decreased with increasing particle size (Figure 4B). Comparing Figure 4A,B one must keep in mind that even if a much lower number of larger particles was delivered to the trabecular meshwork, their overall particle volume was much higher.

### 3.4. Distribution Pattern of Gold NPs in the Outflow Tissue

**Figure 5:**
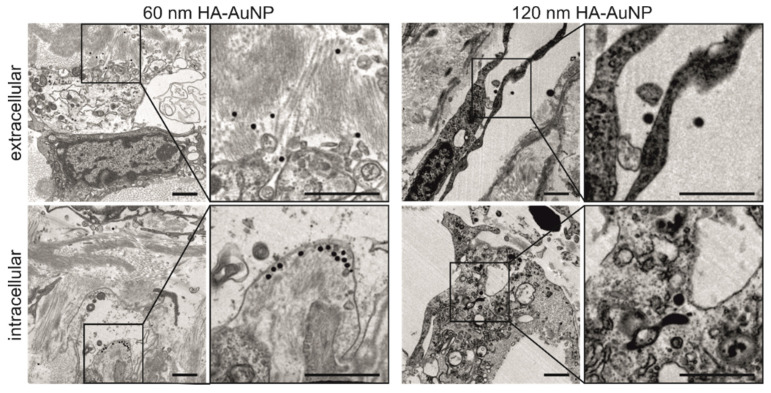
NP distribution within the trabecular meshwork after ex vivo perfusion with gold NPs. Both the 60 and 120 nm HA-AuNPs were located within the trabecular meshwork. Extracellular NPs were found in the extracellular matrix (**upper panel**, **overview image left**) and in the fluid egress pathway (**upper panel**, **magnification image right**). Intracellular NPs were detected in trabecular meshwork cells. Right-hand images are enlargements of the indicated areas of the left-hand images; scale bars represent 1 µm.

Thereafter, the distribution of 60 and 120 HA-AuNPs within the trabecular meshwork was carefully investigated by TEM (Figure 5). Both particle species showed an extracellular and intracellular distribution pattern. Extracellular NPs were detected within the extracellular matrix (Figure 5 upper left panel) and in the so called “open spaces” of the fluid egress pathways (Figure 5 upper right panel). Intracellular NPs were only observed in trabecular meshwork cells, but not in endothelial cells of the aqueous plexus. The porcine aqueous plexus is the anatomical correlate to the human Schlemm´s canal.

The quantification of the NPs showed a uniform distribution in the entire trabecular meshwork (Table 1). The comparison of distribution in the different regions revealed no significant differences between both particle sizes. In addition, both NP sizes were found intracellular in trabecular meshwork cells, in a similar amount (Figure 5). A difference between both particle sizes was that the 120 nm HA-AuNPs were rather taken up by cells in the outer trabecular meshwork, while the 60 nm HA-AuNPs were also taken up by cells in the inner trabecular meshwork. An interesting observation was that nearly all HA-AuNPs remained single in the tissue and did not aggregate (Table 1 and Supplementary Material Figure S3).

### 3.5. Cellular Uptake of Gold NPs In Vitro

To test if a certain gold NP size is also preferably taken up by their target cells, we performed cellular uptake studies. Cultured cells from the trabecular meshwork and Schlemm´s canal were tested (Table 2). Due to its convoluted structure, it was technically not possible to isolate the aqueous plexus after ex vivo perfusion. Nevertheless, we included human Schlemm´s canal cells for in vitro studies due to their importance for glaucoma development and progression [26]. HTM-N cells were used as a trabecular meshwork cell line. In addition, primary hTM cells from two different donors were used. According to the literature, human umbilical vein endothelial cells (HUVECs) were used as surrogate for Schlemm´s canal cells [27,28]. In addition, primary human cells of the Schlemm´s canal (SC) from two different donors were applied. Last but not least, primary human fibroblasts served as a disease model for glaucomatous cells due to the fact that cells in the trabecular meshwork increasingly acquire the phenotype of contractile myofibroblasts during glaucoma development [14]. 

**Figure 6:**
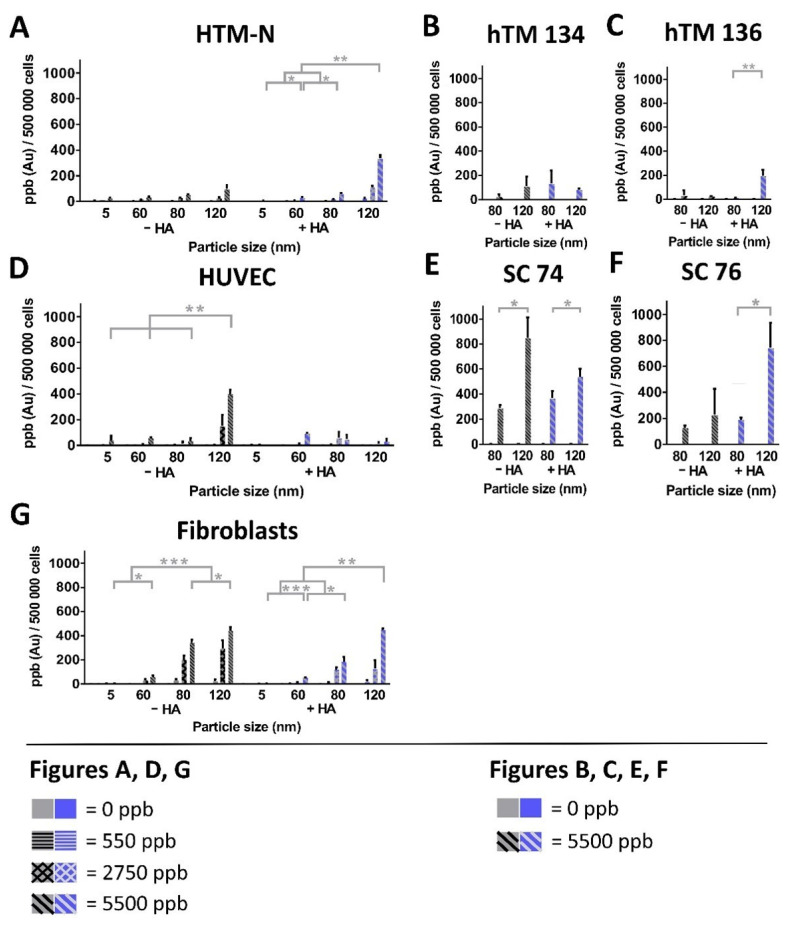
Uptake of AuNPs and HA-AuNPs into different cell types. Different cell types were used to determine the amount of uptake of AuNPs versus HA-AuNPs. Grey bars represent the AuNPs, and blue bars represent the HA-AuNPs. For HTM-N cells, HUVECs and fibroblasts (**A**,**D**,**G**), three different NP concentrations were applied (550, 2750 and 5500 ppb). For primary hTM and SC cells (**B**,**C**,**E**,**F**), only the highest NP concentration was evaluated (5500 ppb). Larger particles seemed to increase the uptake of gold. (**A**) For HTM-N cells, the gold content increased with the NP concentration and the uptake of HA-AuNPs was tendentially higher compared to AuNPs. (**B**) Primary hTM134 cells did not exhibit any significant tendency between the different particle species. (**C**) Primary hTM136 cells demonstrated a higher uptake for the 120 nm HA-AuNPs. (**D**) HUVEC cells showed a lower uptake of HA-AuNPs compared to AuNPs. The 120 nm AuNPs were taken up significantly better than smaller AuNPs. (**E**) Primary SC74 cells displayed higher uptake rates for 120 nm AuNPs or HA-AuNPs, compared to the corresponding 80 nm particles, respectively. (**F**) Primary SC79 cells showed a higher uptake of 120 nm HA-AuNPs compared to 80 nm HA-AuNPs. (**G**) Fibroblasts featured a tendency to have larger particles for both surface modifications and several particle sizes. Statistically significant differences are denoted by *, ** or *** (*p* < 0.05, *p* < 0.01 or *p* < 0.001, respectively).

The four different sizes of gold NPs were added to the cells at three different concentrations for 6 h. In the case of primary cells of the trabecular meshwork and Schlemm´s canal, only 80 and 120 nm gold NPs at the highest concentration were evaluated. The cellular gold content was measured by using ICP-MS (Figure 6). The gold content correlated again to the particle volume. In the trabecular meshwork cell line, the gold content increased with the size and the concentration of the NPs for both AuNPs and HA-AuNPs (Figure 6A). In hTM cells, this trend was not as clear (Figure 6B,C). In HUVECs that served as a surrogate for cells of the Schlemm´s canal, the cellular gold content increased with the size and concentration of AuNPs, but not in the case of HA-AuNPs (Figure 6D). In contrast, when using primary SC cells, the cellular gold content increased with the size irrespective of the surface modification (Figure 6E,F). The fibroblast also nicely followed the pattern of a higher gold content of the cells with increasing particle size and concentration (Figure 6G). 

Finally, the safety of the NPs is of utmost importance for possible therapeutic application. Therefore, the toxicity of the NPs was evaluated in an MTT assay and clearly demonstrated no significant adverse effects for all applied particle types (see Supplementary Material Figure S4).

## 4. Discussion

Glaucoma is a leading cause of acquired blindness and is linked to pathologic changes in the trabecular meshwork and Schlemm´s canal [29]. For successful glaucoma treatment and to avoid adverse events, it will be of utmost importance to deliver therapeutic agents effectively and selectively to the trabecular meshwork. NPs may be an optimal tool for drug delivery to the anterior eye [30], particularly when the delivery of high molecular weight drugs is envisioned [10,17]. Because it is not known which NP size is optimal for this purpose, in this study, we systematically analyzed the uptake and distribution of AuNPs of different surface modifications (unmodified vs. hyaluronic acid) and sizes (5, 60, 80 and 120 nm) in the anterior chamber of the eye.

NPs for drug delivery to the anterior eye can be composed of different materials. As yet, fluorescently labelled PLGA NPs [10] and functionalized silica NPs [17] have been applied for intracameral injection. Polymeric NPs have the advantage that they can be tailored to be biodegradable [31]. In contrast, inorganic NPs are usually not degradable [32]. We hypothesize that NPs that leave the anterior chamber will enter the venous circulation via Schlemm´s canal. Depending on their size, the NPs will then be eliminated from the circulation via the kidney or by the mononuclear phagocyte system [33]. We chose gold NPs as a model in this study because of several advantages, including their easy surface modification using thiol-gold chemistry, availability in different sizes, high biocompatibility with cells and tissues [34] and low detection limits using ICP-MS. Spherical and not rod-shaped AuNPs were used in this study because the results may then be better transferred to lipid or polymer NPs that also have a spherical shape. Different methods were chosen for the characterization of the particles because each one provided different information about their physico-chemical properties. The increase in the particle size was nicely corroborated by UV-Vis spectroscopy, which revealed a right shift of the SPR maximum with increasing particle size. This right shift was expected due to the fact that optical absorption and scattering is dependent on particle size and is described theoretically by Mie theory [25]. Because the SPR maximum also changes with the dielectric constant of the surrounding medium, one could have expected a difference between the coated and uncoated particles of the same size. However, this was only possible for the 5 nm particles. For other particle sizes, this difference was not detected, most likely because the SPR peak broadens with increasing particle size and then the small differences between AuNPs and HA-AuNPs cannot be detected reliably anymore. However, with the help of dynamic light scattering, the expected size increase of AuNPs after coating with HA was clearly demonstrated. At the same time, the PDI of the gold NPs was reduced after coating with HA, indicating a separation of the particles due to shielding effects [20]. 60, 80 and 120 nm particles had a PDI of 0.2 or even lower, accounting for a narrow size distribution of the particles, especially after HA coating. The PDI is calculated as the square of the absolute standard deviation divided by the mean particle diameter. Consequently, size variations cause a disproportionately higher PDI for smaller particles. The lower PDI of the HA-AuNPs might indicate a protective effect of HA against aggregation. The surface zeta potential is an important property of NPs as it influences unspecific binding [35]. It was negative for both surfaces of the gold NPs, which was expected because both citrate ions on unmodified AuNPs and HA carry negative charges. 

The colloidal stability is of utmost importance for NP application. A lack of colloidal stability leads to aggregation of NPs. Consequently, decisive properties of the particles, such as cellular uptake, toxicity or biodistribution, can be altered significantly [36]. The consequence of protein adsorption on the surface of NPs cannot be easily predicted because it may influence the colloidal stability in either a positive or negative manner [37]. In contrast, a high ionic strength of the surrounding media usually negatively influences the colloidal stability [38]. After incubation in cell culture medium containing 0.35% serum, which is equal to the concentration of proteins in the aqueous humor [39], HA-AuNPs did not show any sign of aggregation. In contrast, AuNPs did not withstand these conditions and showed a severe tendency towards aggregation. This indicates a stabilizing effect of HA and is also well known for polyethylene glycol (PEG)-functionalized gold NPs [40]. In contrast, in perfusion medium, there was only a significant increase in the 5 nm AuNPs detectable. This can be attributed to the high specific surface area of these small particles. All other particles were more or less stable, irrespective of their size and surface modification. Generally, bare AuNPs are more stable in phosphate buffer than in serum-containing culture medium [38]. This finding was confirmed by our study, where the unmodified AuNPs demonstrated a higher stability in the perfusion buffer than in the cell culture medium. In addition, the adsorbed proteins to both particle types were semi-quantitatively compared by SDS-PAGE. This brought further evidence for the shielding effects of HA, and because it was clearly demonstrated that the HA coating reduced the protein adsorption significantly on the surface of the gold NPs. 

The perfusion of porcine eyes was performed to study the optimal NP size for targeting the trabecular meshwork. The results clearly illustrate that larger particles deliver a much higher overall particle volume to the trabecular meshwork. All other tissues of the anterior eye showed lower concentrations of gold. Particularly, the cornea and the lens demonstrated exceptionally low gold concentrations and were comparable to the control. This distribution pattern is beneficial for targeting the trabecular meshwork as it minimizes the risk of off target effects, especially to the cornea, which is a natural anatomical barrier and is therefore not expected to take up NPs at a significant level [41]. Studies assessing the ocular biodistribution of topically applied polymeric NPs revealed a relatively low distribution to the iris and the cornea [42]. In addition, if too many NPs end up in the cornea or the lens, it may interfere with vision due to scattering effects [25]. Further in vivo studies will be necessary to confirm and substantiate the results of this study. A potential distribution of NPs from the anterior chamber to the retina and/or vitreous body seems highly unlikely due to the physiology of the aqueous humor dynamics [43] and was therefore not investigated in this study. In fact, the velocity of posterior fluid movement is negligible compared to the aqueous humor flow in the anterior chamber [44]. Therefore, drugs applied to the anterior chamber are primarily eliminated through the aqueous humor [45,46]. For targeting the trabecular meshwork, larger particles regardless of their surface showed favorable properties. Unfortunately, unmodified 5 nm AuNPs were prone to aggregation and therefore acted like larger particles. The uncontrolled tendency towards aggregation makes them useless for drug delivery purposes. Even though there was no difference between the AuNPs and HA-AuNPs for 60, 80 and 120 nm particles, one would choose HA-AuNPs for therapeutic application due to their significantly higher colloidal stability. Electron microscopy of tissue samples corroborated that HA coating is beneficial because even in the trabecular meshwork the particles remained single and did not aggregate. In our in vitro and ex vivo experiments, the measured gold concentration directly correlated to the particle volume. Consequently, larger gold NPs lead to a higher overall particle volume delivered to the trabecular meshwork. Generally, active pharmaceutical ingredients can either be attached to the surface of NPs or encapsulated into the particles’ matrix. Our findings are particularly important for NPs with encapsulated drugs because a higher particle volume offers much more space for the drug and allows for a much higher drug content per particle. Consequently, even if fewer larger particles have arrived at the target tissue, they would lead to an overall higher drug amount. 

The ex vivo perfusion experiments confirmed that the NPs preferentially arrived in the target tissue in a high amount and that the 120 nm particles showed a slight superiority compared to the smaller sizes Many drugs, such as siRNA, have their place of action inside cells. An intracellular location of the NPs after perfusion was confirmed by TEM. Once arrived at the target cells, NPs have the great advantage to allow for an efficient cellular uptake of their therapeutic freight [8]. Macromolecular drugs in particular, such as siRNA, would otherwise be highly unstable and not be able to cross cellular membranes [9]. In a next step, we substantiated and quantified the cellular uptake in vitro. In addition to trabecular meshwork cells, we also chose cells of Schlemm´s canal, because these cells are equally important for disease development and progression [47]. The trend of a higher uptake of larger particles was demonstrated in most, but not all cell types. It was very remarkable that primary cells showed an efficient uptake of NPs and that particularly Schlemm´s cells showed the highest uptake rate amongst the primary cell types, which is of utmost importance for drug delivery. Another fact of importance was the favorable uptake of gold NPs into fibroblasts. Trabecular meshwork cells show a transformation to myofibroblasts in the course of the disease [14]. According to our experiments, this transformation will not impede NP uptake. However, one must keep in mind that at late-stage glaucoma there is a significant loss of trabecular meshwork cells [48]. For application of NPs that are envisioned to target the outflow tissues, two options are conceivable. The NPs are injected into the anterior chamber of the eye, thereby avoiding the significant barrier of the cornea. Regarding this application route, there is only limited literature and clinical experience so far compared to intravitreal injections [4,49]. However, because drug delivery devices for intracameral injection are gaining increasing attention [50,51], guidelines on intracameral injection techniques have been provided recently [49]. Another limitation is that frequent application intervals would be necessary because the aqueous humor has a rapid turnover [4]. To cope with this shortcoming, a depot formulation containing the NPs would be ideal in the long run. Such a depot would be applied intraocularly, for example, into the vitreous body [52]. After release from the depot, the NPs would reach the anterior chamber and be distributed to the trabecular meshwork. Such a formulation would be more patient friendly and most likely associated with a much higher compliance because it would reduce the application frequency dramatically. Another location for a depot formulation would be the supraciliary space or the anterior chamber [4].

## 5. Conclusions

We successfully investigated the relevance of NP size for targeting the trabecular meshwork after intracameral injection ex vivo. Particles with a diameter of 120 nm exhibited the highest volume-based accumulation rate in the trabecular meshwork, while smaller particles were superior regarding the delivered particle number. This fact is useful for drug delivery as it enables particles to selectively target the trabecular meshwork by choosing the optimal size and thereby reducing off-target effects. Our results additionally suggest that NPs of about 120 nm can be a useful tool for glaucoma therapy due to their higher drug encapsulation capacity. Consequently, a lower number of particles will be needed to elicit a therapeutic effect.

## Figures and Tables

**Table 1:** Quantitative evaluation of distribution of 60 or 120 nm HA-AuNPs in TEM images. Three independent experiments were conducted. TM = trabecular meshwork.

Criteria	60 nm	120 nm	Statistics
particles per 1000 µm² –of total TM (±SEM) –of outer TM (±SEM) –of inner TM (±SEM)	1.18 ± 0.75 1.55 ± 1.12 1.02 ± 0.14	1.71 ± 0.62 1.05 ± 0.61 2.12 ± 0.87	*p* = 0.61 *p* = 0.66 *p* = 0.37
aggregated NPs	0 of 125	2 of 73	

**Table 2:** Cell types used for the cellular uptake of NPs in vitro.

Tissue/Condition	Immortalized Cell Lines	Primary Cells
trabecular meshwork	HTM-N	hTM (no. 134 and 136)
endothelial cells	–	SC (no. 74 and 79) HUVEC
disease model	–	fibroblasts

## Data Availability

Not applicable.

## Supplementary Materials

The following are available online at https://www.mdpi.com/article/10.3390/pharmaceutics13060901/s1, Figure S1: ^1^H-NMR-spectra of hyaluronic acid and thiolated hyaluronic acid in comparison, Figure S2: Determination of protein adsorption by using SDS-PAGE, Figure S3: Aggregated HA-AuNPs, Figure S4: MTT-Assay to determine the cell toxicity.
