# Supplementary material for: Distribution of Gold Nanoparticles in the Anterior Chamber of the Eye after Intracameral Injection for Glaucoma Therapy
Source: Pharmaceutics. 2021 Jun 17;13(6):901. doi: 10.3390/pharmaceutics13060901 (PMC8235414; doi:10.3390/pharmaceutics13060901)
Supplement: Supplementary file 1 [file pharmaceutics-13-00901-s001.zip › pharmaceutics-1249338-supplementary.pdf]

pharmaceutics

MDPI

Supplementary Materials: Distribution of Gold Nanoparticles in the Anterior Chamber of the Eye after Intracameral Injection for Glaucoma Therapy

Tobias Sonntag, Franziska Froemel, W. Daniel Stamer, Andreas Ohlmann, Rudolf Fuchshofer, Miriam Breunig

S1: ^{1}H-NMR Spectra of Hyaluronic Acid (HA) and Thiolated Hyaluronic Acid (HA-SH)

HA-SH was characterized using a Avance 300 NMR-spectrometer (Bruker Bio Spin, Ettlingen, Germany). ^{1}H-NMR spectra of HA and HA-SH in Deuterium oxide were compared and revealed an additional peak at δ=3.2 ppm, indicating a successful modification of HA.

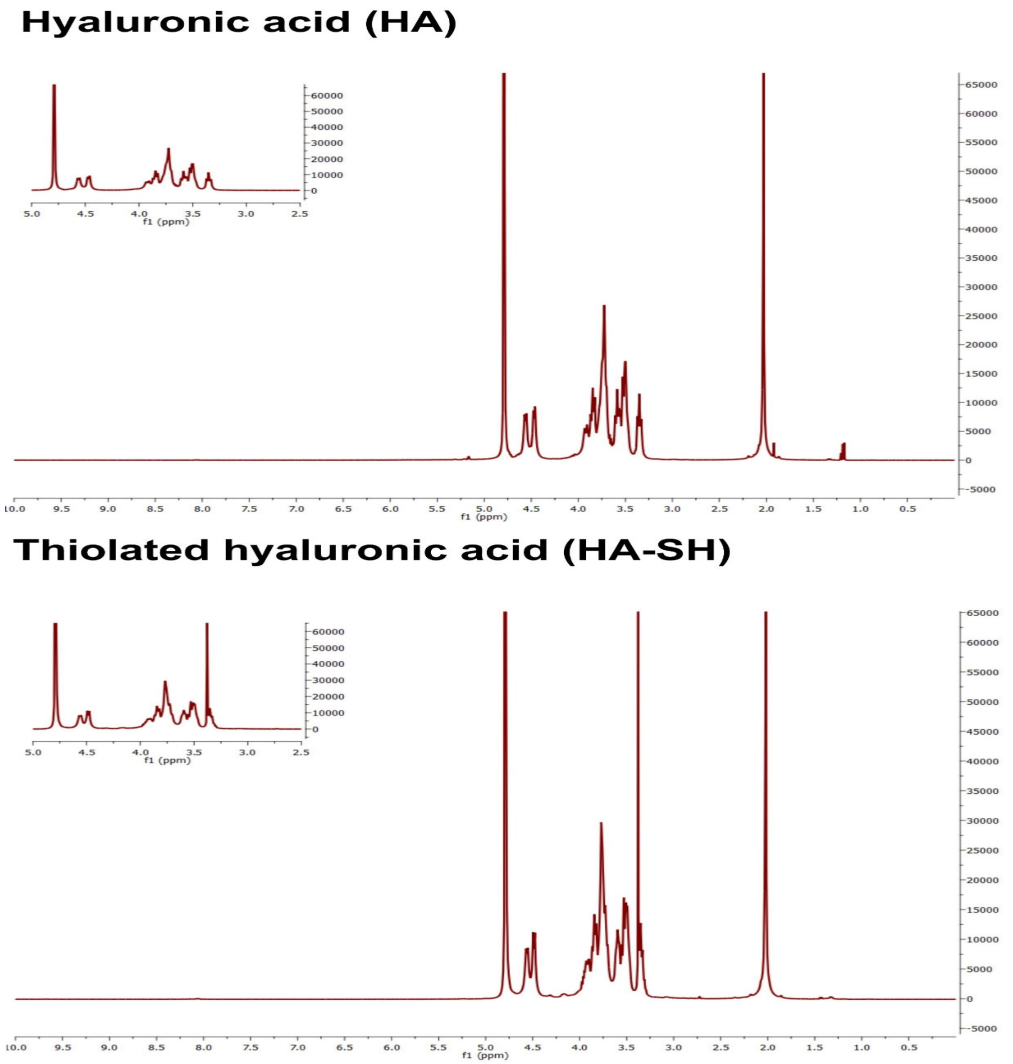

Figure S1. ^{1}H-NMR spectra of HA and HA-SH in comparison. ^{1}H-NMR-spectra indicated the successful synthesis of HA-SH due to the additional peak at δ=3.2 ppm in the spectrum of HA-SH.

S2: Analysis of Protein Adsorption

Incubation of nanoparticles in a physiological environment will lead to protein adsorption on the surface [1]. This is of utmost importance because protein adsorption significantly influences the colloidal stability as well as the interaction with potential target cells [1]. To evaluate protein adsorption, AuNPs and HA-AuNPs were incubated with serum containing culture medium. Particles were washed and afterwards an SDS-PAGE of washed particles was performed. Proteins that were adsorbed to the nanoparticle surface will be peeled off in an electric field and separated according to their molecular weight. As a control the same amount of serum without particles was used. The bands arising from the HA-AuNPs were much fainter and nearly invisible compared to AuNPs. Therefore, in direct comparison it became evident that a much lower number of proteins adsorbed to HA-AuNPs.

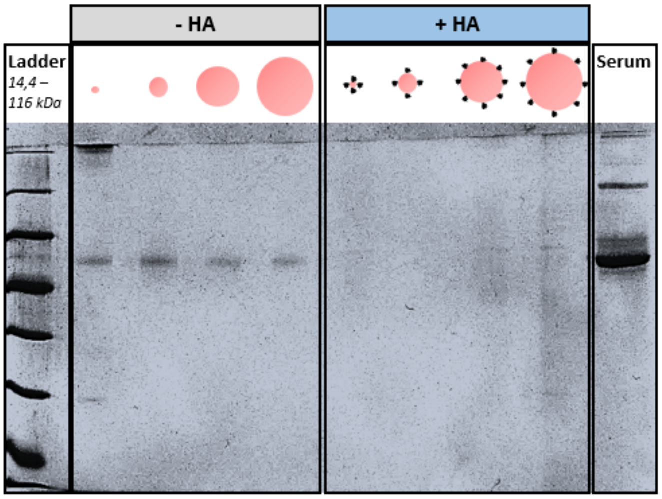

Figure S2. Determination of protein adsorption by using SDS-PAGE. Particles at a concentration of c(Au)=18,000 ppb were incubated in culture medium. After 1 h of incubation and several washing and centrifugation steps an SDS-PAGE was performed. On the left side of the figure a protein ladder was applied to the gel. On the right side a serum control was applied. A comparison of the spots of unmodified particles (-HA) and modified particles (+HA) reveals a significant protein adsorption of serum to the surface of the AuNPs.

S3: Electron Microscopic Analysis of Nanoparticle Distribution and Aggregation Within the Trabecular Meshwork

Investigating the distribution pattern of 60 and 120 nm HA-AuNPs, only two 120 nm HA-AuNP were found to be in immediate contact.

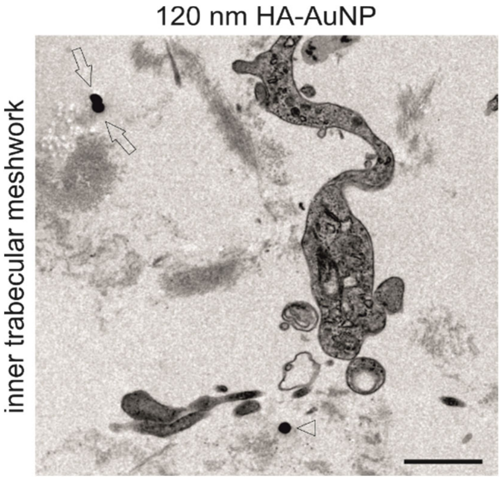

Figure S3. Nanoparticle aggregation within the trabecular meshwork after ex vivo perfusion with gold nanoparticles. The only two aggregated HA-AuNP found in this study (arrows indicate aggregated NPs, arrowhead indicates individual NP, scale bar represents 1 μm).

S4: MTT-Assay of AuNPs and HA-AuNPs

Cytotoxicity of all nanoparticle types was assessed using an MTT-assay (MTT=3-(4,5-dimethylthiazol-2-yl)-2,5-diphenyltetrazolium bromide). The assay was performed according to ISO 10993-5:2009 [2] (Biological evaluation of medical devices, part 5: Tests for in vitro cytotoxicity). HTM-N cells were seeded in 96-well plates at a density of 10,000 cells per well and allowed to adhere for 12 h. Every particle type was pre-incubated with cell culture medium containing 0.35% (*v*/*v*) FBS for 1 h. The pre-incubation was performed in the final medium containing a gold concentration of concentration 5500 ppb for every particle type. 100 μL nanoparticles dispersed in cell culture medium was added to each well. SDS (0.1% (*v*/*v*) served as a negative control and pure culture medium as a positive control (n=6). Cells were incubated with the test media for 24 h, afterwards the medium was removed and 200 μL of serum-containing medium supplemented with 1.5 mM MTT was added to each well. After 4 h of incubation, the MTT solution was removed and 100 μL of DPBS containing 10% SDS (*v*/*v*) was added to each well. After 14 h of incubation, absorbance at 570 and 690 nm was determined for each well using a FluoStar Omega fluorescence microplate reader (BMG Labtech, Ortenberg, Germany). The difference in absorbance at λ=570 and λ=690 nm was used to calculate the cell viability. For normalization, the viability of control cells was set at 100%.

In the in vitro experiments, the incubation time of the particles with the cells was set to 6 h. For the MTT-assay, the cells were incubated for a significantly longer time period of 24 h with the nanoparticles. Even after this prolonged incubation at the highest particle concentration used for cell experiments the cell viability was at least 80% or even higher. This strongly indicates that there is no cytotoxicity.

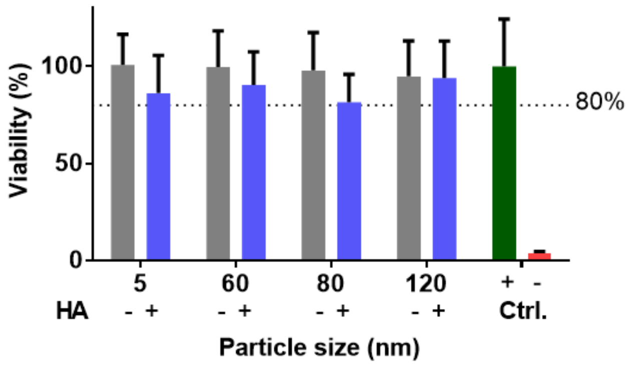

Figure S4. MTT-Assay to determine the cell toxicity. An MTT-assay was performed for all particle types using HTM-N cells. In all cases, the cell viability was at least 80% after 24 h. Positive and negative control (Ctrl.) show the expected results.

References

1. C.D. Walkey, J.B. Olsen, H. Guo, A. Emili, W.C.W. Chan, Nanoparticle size and surface chemistry determine serum protein adsorption and macrophage uptake, *J. Amer. Chem. Soc*. **2012**, *134*, 2139–2147.
2. M. Gregoritza, V. Messmann, K. Abstiens, F.P. Brandl, A.M. Goepferich, Controlled Antibody Release from Degradable Thermoresponsive Hydrogels Cross-Linked by Diels-Alder Chemistry, *Biomacromolecules* **2017**, *18*, 2410–2418.
